# Effects of a Common Eight Base Pairs Duplication at the Exon 7-Intron 7 Junction on Splicing, Expression, and Function of OCT1

**DOI:** 10.3389/fphar.2021.661480

**Published:** 2021-05-07

**Authors:** Sarah Römer, Marleen J. Meyer, Kathrin Klein, Lennart V. Schneider, Johannes Matthaei, Ana Tzvetkova, Joanna Łapczuk-Romańska, Jochen Gaedcke, Marek Droździk, Jürgen Brockmöller, Anne T. Nies, Mladen V. Tzvetkov

**Affiliations:** ^1^ Institute of Pharmacology, Center of Drug Absorption and Transport (C_DAT), University Medicine Greifswald, Greifswald, Germany; ^2^ Dr. Margarete Fischer-Bosch Institute of Clinical Pharmacology, Stuttgart, Germany; ^3^ University of Tuebingen, Tuebingen, Germany; ^4^ Institute of Clinical Pharmacology, University Medical Center Göttingen, Göttingen, Germany; ^5^ Institute of Bioinformatics, University Medicine Greifswald, Greifswald, Germany; ^6^ Human Molecular Genetics Group, Department of Functional Genomics, Interfaculty Institute of Genetics and Functional Genomics, University Medicine Greifswald, Greifswald, Germany; ^7^ Department of Experimental and Clinical Pharmacology, Pomeranian Medical University, Szczecin, Poland; ^8^ Department of General, Visceral and Pediatric Surgery, University Medical Center Göttingen, Göttingen, Germany; ^9^ Cluster of Excellence iFIT (EXC2180) “Image-Guided and Functionally Instructed Tumor Therapies”, University of Tuebingen, Tuebingen, Germany

**Keywords:** ins/del variant, organic cation transporter 1, SLC22A1, minigene, allelic expression imbalance (AEI), fenoterol, sumatriptan, pharmacokinetics

## Abstract

Organic cation transporter 1 (OCT1, SLC22A1) is localized in the sinusoidal membrane of human hepatocytes and mediates hepatic uptake of weakly basic or cationic drugs and endogenous compounds. Common amino acid substitutions in OCT1 were associated with altered pharmacokinetics and efficacy of drugs like sumatriptan and fenoterol. Recently, the common splice variant rs35854239 has also been suggested to affect OCT1 function. rs35854239 represents an 8 bp duplication of the donor splice site at the exon 7-intron 7 junction. Here we quantified the extent to which this duplication affects OCT1 splicing and, as a consequence, the expression and the function of OCT1. We used pyrosequencing and deep RNA-sequencing to quantify the effect of rs35854239 on splicing after minigene expression of this variant in HepG2 and Huh7 cells and directly in human liver samples. Further, we analyzed the effects of rs35854239 on OCT1 mRNA expression in total, localization and activity of the resulting OCT1 protein, and on the pharmacokinetics of sumatriptan and fenoterol. The 8 bp duplication caused alternative splicing in 38% (deep RNA-sequencing) to 52% (pyrosequencing) of the minigene transcripts when analyzed in HepG2 and Huh7 cells. The alternatively spliced transcript encodes for a truncated protein that after transient transfection in HEK293 cells was not localized in the plasma membrane and was not able to transport the OCT1 model substrate ASP^+^. In human liver, however, the alternatively spliced OCT1 transcript was detectable only at very low levels (0.3% in heterozygous and 0.6% in homozygous carriers of the 8 bp duplication, deep RNA-sequencing). The 8 bp duplication was associated with a significant reduction of OCT1 expression in the human liver, but explained only 9% of the general variability in OCT1 expression and was not associated with significant changes in the pharmacokinetics of sumatriptan and fenoterol. Therefore, the rs35854239 variant only partially changes splicing, causing moderate changes in OCT1 expression and may be of only limited therapeutic relevance.

## Introduction

OCT1 (SLC22A1) is by far the most strongly expressed transporter of organic cations in the sinusoidal membrane of the human liver (Nies et al., 2009; Wang et al., 2015; Drozdzik et al., 2019). OCT1 mediates the first step of hepatic clearance of weakly basic or positively charged drugs. Metformin, morphine, sumatriptan or fenoterol and endogenous compounds like thiamine belong to substrates transported by OCT1 (Wang et al., 2002; Tzvetkov et al., 2013; Chen et al., 2014; Matthaei et al., 2016; Tzvetkov et al., 2018). A loss of OCT1 function was shown to increase plasma concentrations of several drugs including sumatriptan and fenoterol (Kerb et al., 2002; Shu et al., 2007; Tzvetkov et al., 2013; Arimany-Nardi et al., 2016; Matthaei et al., 2016; Tzvetkov et al., 2018). Depending on administered drugs, an increase may bear the risk of toxic side effects and may affect drug efficacy.

OCT1 is encoded by the SLC22A1 gene, which is located on the long arm of human chromosome 6 (6q26) and contains 11 exons and 10 introns (Koehler et al., 1997; Zhang et al., 1997). The resulting OCT1 protein has 554 amino acids and is composed of 12 transmembrane helices (TMHs) with intracellularly localized N- and C-termini.

The SLC22A1 gene shows the highest genetic variability within the pharmacologically relevant members of the SLC22 family (Tzvetkov et al., 2016; Schaller and Lauschke, 2019). Fourteen single nucleotide polymorphisms (SNPs) result in amino acid substitutions. Thereof, four common amino acid substitutions (Arg61Cys, Cys88Arg, Gly401Ser, and Gly465Arg) and a deletion of Met420 are known to confer strongly reduced or completely abolished OCT1 activity (Kerb et al., 2002; Shu et al., 2003; Shu et al., 2008; Seitz et al., 2015). Nine percent of Europeans and White Americans are homozygous or compound heterozygous carriers of these reduce function variants (Seitz et al., 2015). These individuals (also referred to as poor OCT1 transporters) have significantly altered pharmacokinetics resulting in altered efficacy and toxicity of clinically relevant drugs like sumatriptan, fenoterol, tramadol and morphine (Shu et al., 2008; Becker et al., 2011; Tzvetkov et al., 2011; Tzvetkov et al., 2012; Fukuda et al., 2013; Tzvetkov et al., 2013; Stamer et al., 2016).

Non-coding variants may also affect OCT1 activity, e.g. by altering OCT1 expression. Indeed, SLC22A1 expression varies strongly between individuals (Nies et al., 2009; O’Brien et al., 2013). The OCT1 mRNA levels differ up to 113-fold and protein levels up to 83-fold between individuals (Nies et al., 2009). However, common promoter variants did not significantly affect the SLC22A1 promoter activity or mRNA expression (Bokelmann et al., 2018).

**FIGURE 1:**
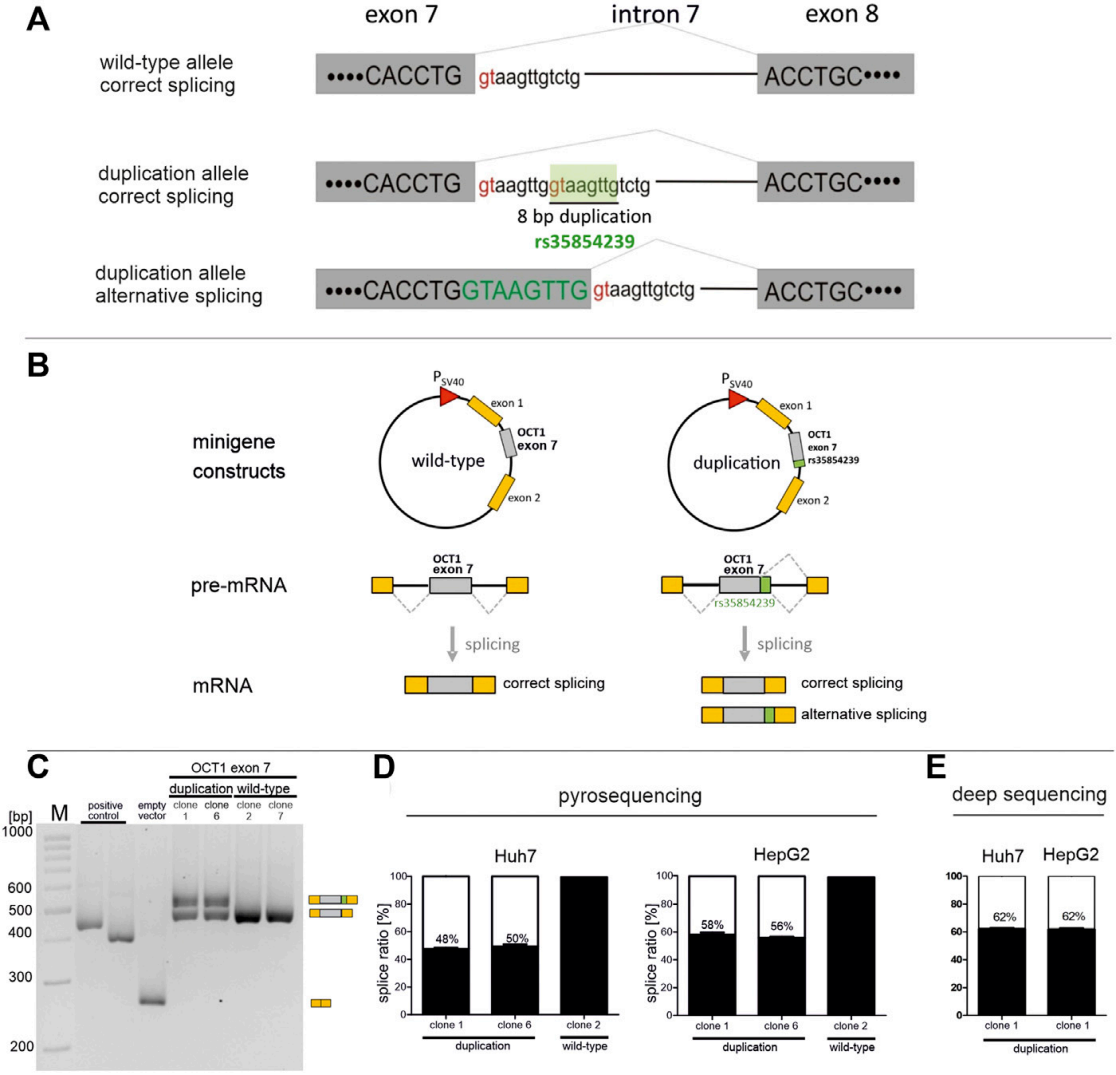
Effects of rs35854239 on SLC22A1 exon 7 minigene splicing in Huh7 and HepG2 **(A)** Splicing at the exon 7-intron 7 junction. Splice donor sites within the intronic sequence are shown in red. The 8 bp insertion/deletion variant rs35854239 carries a second splice donor site that is proposed to be spliced alternatively. **(B)** Representation of the pSPL3b splicing vector consisting of two exons of the rabbit β-globulin gene under control of the SV40 promoter (the “minigene”). SLC22A1 exon 7 and its flanking intronic regions with or without rs35854239 (referred to as duplication or wild-type, respectively) were cloned between both exons of the rabbit β-globulin gene. Minigene constructs were transiently transfected into Huh7 and HepG2 cells, and mRNA was isolated 48 h after transfection. As positive control we used the CYP2C19*2 variant, for which alternative splicing is known, (Morais et al., 1994). **(C,D)** Correctly and alternatively spliced OCT1 transcripts were shown **(C)** and quantified using pyrosequencing **(D)**, or deep RNA-sequencing **(E)**. Percentages within boxes represent relative values of correctly spliced minigene transcripts. Data are shown as mean and standard errors of the mean of at least three independent experiments.

Another explanation of the high variability in OCT1 expression may be related to genetic variants that cause alternative splicing. Indeed, an 8 base pairs insertion/deletion variant rs35854239 (formerly also designated as rs113569197 or rs36056065) was suggested to affect OCT1 expression and activity by altering splicing (Tarasova et al., 2012; Grinfeld et al., 2013; Kim et al., 2017). This variant is located at the junction of exon 7 and intron 7 of the SLC22A1 gene and represents an 8 bp duplication of the 5’ part of intron 7 including the splice donor site (Figure 1A). The newly generated donor site results in an 8 bp longer transcript with shift in the open reading frame and a premature stop-codon.

The rs35854239 variant is genetically highly linked to the coding variant Met408Val (*r* ^2^ of 0.95). In several studies, Met408Val was associated with drug efficacy. This association has been explained by decreasing cellular uptake and thus altering the systemic concentrations or concentrations at the site of action of the drug. However, multiple independent *in vitro* studies demonstrated that the Met408Val substitution does not directly affect OCT1 uptake (Kerb et al., 2002; Shu et al., 2003; Shu et al., 2007; Nies et al., 2014; Tzvetkov et al., 2014; Seitz et al., 2015). Thus, the rs35854239 variant may be the true cause for the observed associations of Met408Val with clinically relevant phenotypes.

The rs35854239 variant is very common. If functional, with its minor allele frequency of 40.6% in Europeans and White Americans, the rs35854239 variant could be the most frequent variant affecting OCT1 expression and activity. However, it is not clear whether the duplicated splicing donor site always leads to alternative splicing, and to what extend the alternatively spliced transcript is functionally active.

In this study, we analyzed to what extend the SLC22A1 8 bp duplication (rs35854239) affects splicing and what are the consequences of the variable splicing on the transporter function *in vitro* and *in vivo*. To this end, first, we quantified the alternatively spliced transcripts both using the minigene assay and direct analyses of human liver samples. Second, we analyzed whether the protein resulting from the alternatively spliced transcripts is active. Finally, we analyzed whether the rs35854239 variant is associated with changes in OCT1 mRNA and protein expression in human livers and pharmacokinetics of sumatriptan and fenoterol in humans.

## Materials and Methods

### Materials

Dulbecco’s Modified Eagle Medium (DMEM), Hank’s Buffered Salt Solution (HBSS), and 4-(4-(dimethylamino)styryl)-N-methylpyridinium (ASP^+^) were obtained from Life Technologies (Darmstadt, Germany). Poly-D-lysine (1–5 kDa), 2-[4-(2-hydroxyethyl)piperazin-1-yl]ethanesulfonic acid (HEPES), bicinchoninic acid, and copper sulfate pentahydrate were obtained from Sigma-Aldrich (Taufkirchen, Germany). Dulbecco’s phosphate-buffered saline (DPBS) and additives for cell culturing were obtained from PAN-Biotech (Aidenbach, Germany). Twelve-well plates were obtained from CytoOne (Langenselbold, Germany), 6-well plates from Corning GmbH (Kaiserslautern, Germany), and tissue culture flasks from Sarstedt (Nümbrecht, Germany). All chemicals used in this study were purchased from commercial sources and had purities ≥95%.

### Cell Lines and Cell Culturing

HEK293 (Thermo Fisher Scientific, Darmstadt, Germany), Huh7, and HepG2 (ATCC, Manassas, United States) cells were cultured in DMEM supplemented with 10% fetal calf serum (FCS), 100 U/mL penicillin and 100 μg/ml streptomycin at 37°C and 5% CO_2_.

### Generation of an Alternatively Spliced OCT1 Plasmid

The 8 bp insertion in exon 7 that results from alternative splicing of rs35854239 was introduced into an OCT1 encoding pcDNA5/FRT vector by site-directed mutagenesis as described previously (Seitz et al., 2015). The used primer pair 1 is listed in Supplementary table S1. The sequence was validated by capillary sequencing prior to transient transfection into HEK293 cells.

### Cellular Uptake Experiments After Transient Transfection

HEK293 cells were seeded at a density of 5 × 10^5^ cells per well in a 12-well plate precoated with poly-D-lysine. Twenty-four hours after seeding, cells were transfected with 2 µg of the alternatively spliced OCT1 vector DNA using Lipofectamine™ 2000 (Invitrogen, Darmstadt, Germany) according to the manufacturer’s instructions. Transfection efficiency was evaluated by co-transfection with 0.5 µg of the green fluorescent protein coding vector pGFP-tpz (Thermo Fisher Scientific). The next day, uptake experiments were performed at 37°C and pH 7.4 using HBSS+ (HBSS supplemented with 10 mM HEPES buffer). Cells were washed once with pre-warmed (37°C) HBSS+. Uptake was initiated by adding 20 µM ASP^+^ diluted in HBSS+ and stopped after two minutes by adding ice-cold HBSS+. Cells were washed twice with ice-cold HBSS+ and lyzed with RIPA buffer. Fluorescence of ASP^+^ in lysates was measured with an excitation of 485 nm and emission of 612 nm using the Tecan infinite M200 Microplate Reader (Tecan Group Ltd., Männedorf, Switzerland). ASP^+^ fluorescence intensities were normalized to the total protein amount in the samples as measured using the bicinchoninic acid assay (Smith et al., 1985).

### Generation of Minigene Constructs

Splicing of exon 7-intron 7 was analyzed in the splicing vector pSPL3b, further referred to as minigene. Exon 7-intron 7 of OCT1 for both rs35854239 genotypes was amplified with primer pair 2, listed in Supplementary table S1. The PCR product was cloned into the pSPL3b vector after restriction of the PCR product and the vector with *Pst*I and *Eco*RV. The Met408 and Val408 were introduced by site-directed mutagenesis using primer pairs 3 and 4, respectively, listed in Supplementary table S1. The minigene constructs were validated by capillary sequencing and then used for transient transfection into Huh7 and HepG2 cells.

### Transient Transfection of the Minigene Constructs

Huh7 and HepG2 cells were seeded in six-well plates at a density of 4 × 10^5^ and 1.7 × 10^6^ cells per well, respectively. After 24 hours, cells were transfected with 2 µg minigene vector DNA using Lipofectamine™ 2000 as described above. Transfection efficiency was evaluated by co-transfection with pGFP-tpz as described above. Forty-eight hours after transfection, cells were lysed and RNA was isolated using the RNeasy Plus Mini Kit (QIAGEN, Hilden, Germany) according to the manufacturer’s instructions.

### PCR Amplification of Spliced Exon 7 Variants

After RNA isolation from transfected cells, complementary DNA (cDNA) was synthesized using the MultiScribe™ Reverse Transcriptase Kit (Applied Biosystems). Spliced exon 7 was amplified with primer pair 5 listed in Supplementary table S1. PCR products were separated by gel electrophoresis, bands were visualized under UV light and band intensities were quantified using the Fiji software (ImageJ version 1.52p, National Institutes of Health, Bethesda, United States).

### Analysis of rs35854239 in Human Liver Samples

Human liver samples were obtained from normal liver tissue that had to be removed for technical reasons during liver surgery or from organ donors. Patients gave their informed consent for research use of the removed liver tissue, and the procedures were approved by the ethics committee of the University Medicine Göttingen, Georg-August-Universität Göttingen (application number 26/01/17) and the ethics committee of the Pomeranian Medical University (application number KB-0012/64/12). Deep-frozen human liver samples were homogenized using the Mikro dismembrator S (B. Braun, Melsungen, Germany) at 2500 rpm for 1 min. DNA was isolated using the DNeasy Blood & Tissue kit (QIAGEN) according to the manufacturer’s instructions. For the genotyping of functionally relevant polymorphisms in the SLC22A1 gene, the single base primer extension method was used as described previously (Seitz et al., 2015) using primer 6 listed in Supplementary table S1. RNA from human liver samples was isolated from homogenates using the RNeasy Plus Mini Kit and cDNA was synthesized as described above.

### Pyrosequencing

The ratio of correctly vs. alternatively spliced exon 7 in the transfected cell lines with hepatic origin and human liver samples was analyzed using pyrosequencing. Spliced exon 7 from minigene experiments and from cDNA from human liver samples was amplified using primer pair 7 (minigene) and primer pair 8 (liver samples) listed in Supplementary table S1. Samples were prepared using PyroMark™ Binding and Annealing Buffer (QIAGEN) and the PyroMark™ Vacuum Prep Station (Biotage, Uppsala, Sweden). Pyrosequencing was carried out on the PyroMark™ Q96 ID (Pyrosequencing AB, Uppsala, Sweden) with PyroMark™ Gold Q96 reagents (QIAGEN) using the primer 9 (minigene) and primer 10 (liver samples).

### Deep RNA-Sequencing and Sequence Mapping

Next-generation DNA and RNA sequencing was performed with cDNA from minigene experiments and DNA and cDNA from human liver samples. Exon 7 and its 3′ flanking region were first amplified using the primer pairs 11 to 14 listed in Supplementary table S1. The PCR products were purified by magnetic separation using Agencourt^®^ AMPure^®^ XP reagent (Beckman Coulter GmbH, Krefeld, Germany). Unique indices were attached to the purified amplicons by PCR using the Nextera^®^ XT Index Kit v2 (Illumina Inc., San Diego, United States). The samples were again cleaned up with Agencourt^®^ AMPure^®^ XP reagent. All samples were pooled in appropriate ratios. The pooled library was quantified using the Qubit^®^ 2.0 fluorometer and the Qubit^®^ dsDNA BR assay kit (Thermo Fisher Scientific) and diluted to DNA concentration of 2 nM. DNA was denatured and diluted according to the manufacturer’s instructions. As internal control and to increase variability within the sequencing run, 30% PhiX control (Illumina) was spiked in prior to denaturation. The sequencing run was performed using the MiSeq^®^ Reagent Kit v3 (600 cycles) and paired-end 221 reads on the Illumina MiSeq™ (Illumina). The sequencing run was analyzed using the IGV v.2.6.3 software (Broad Institute, Cambridge, United States).

The paired-end sequence reads were merged using PEAR (release 0.9.11; Zhang et al., 2014). The mapping to a reference sequence was performed with Bowtie2 (Langmead et al., 2012) version 2.3.4.1. DNA was mapped against the human genome assembly (hg19) downloaded from the UCSC Genome Browser. The cDNA from human liver samples was mapped against the “Homo sapiens solute carrier family 22 member 1 (SLC22A1), transcript variant 1, mRNA” (NM_003057.3) with artificially introduced duplication in it in order to better visualize the possible insertion using local mapping mode. The cDNA from minigene was mapped against the minigene itself. For both experiments with human liver samples we calculated subsequently the sequencing depth for each allelic combination of rs35854239 variant and rs628031 (Met408Val) variant with our own script.

### Immunocytochemistry

Five x 10^5^ HEK293 cells were seeded on poly-D-lysine coated cover slips and transfected as described above. One day after transfection, cells were washed twice with PBS and were fixed with 100% ethanol for 20 min at –20°C. After washing three times with PBS, cell membranes were permeabilized with PBS containing 0.4% Tween 20. Cells were washed three times with PBS and subsequently blocked for 3 hours with blocking buffer (5% FCS in PBS). OCT1 was stained using the NBP1-51684 (2C5) antibody (Novus Biologicals, Abingdon, United Kingdom). Cells were co-stained with the EP 1845Y antibody (Abcam, Cambridge, United Kingdom) against the membrane marker Na^+^/K^+^-ATPase. The primary antibodies against OCT1 and Na^+^/K^+^-ATPase were diluted in blocking buffer in a dilution of 1:400 and 1:200, respectively. Per cover slip, 50 µL antibody solution was added, cells were covered with parafilm and incubated in a humid chamber overnight. The next day, after washing three times with PBS, fluorescently-labeled secondary antibodies (Alexa Fluor^®^ 546 goat anti-rabbit IgG (H + L), polyclonal and Alexa Fluor^®^ 488 goat anti-mouse IgG (H + L), polyclonal; Thermo Fisher Scientific) were diluted 1:400 in PBS, added and incubated for 2 hours in the dark. Cells were washed three times with PBS and cover slips were mounted with Roti^®^ Mount Fluor Care DAPI (Carl Roth, Karlsruhe, Germany). The staining was analyzed using the laser scanning microscope LSM780 (Carl Zeiss Microscopy GmbH, Oberkochen, Germany). Images were processed using the Fiji software.

### Expression Data From Human Liver Samples

OCT1 mRNA and OCT1 protein expression data were extracted from a previous study describing expression of OCT1 and OCT3 in human liver samples (Nies et al., 2009). Analysis was performed on the subset of samples (*n* = 90) that were from individuals who were non-cholestatic and had no hepatocellular, cholangiocellular or gallbladder carcinoma (Schaeffeler et al., 2011; Nies et al., 2013). The study was approved by the ethics committees of the Charité, Humboldt University (Berlin, Germany) and the University of Tübingen (Tübingen, Germany) in accordance with the principles of the Declaration of Helsinki. Written informed consent was obtained from each patient.

### Clinical Trial

The clinical trials on the effects of SLC22A1 genetic variants on fenoterol and sumatriptan pharmacokinetics have been described in details before (Matthaei et al., 2016; Tzvetkov et al., 2018). The rs35854239 variant was genotyped using the available DNA from those studies and the single base primer extension method as described previously (Seitz et al., 2015) with the SNaPshot primers listed in Supplementary table S1.

### Statistical Analysis

Differences in OCT1 mRNA and protein expression, or drug plasma concentration between homozygous wild-types and homozygous duplication allele carriers were performed using the Mann-Whitney-*U* test. Differences between DNA and RNA allele frequencies in allelic expression imbalance analyses were performed using the paired sample *t*-test. All analyses were performed using SPSS Statistics version 25 (SPSS INC., IBM, Chicago, IL) Statistical significance was defined as *p* < 0.05. Post-hoc power calculations of the clinical studies were performed with the G*Power software version 3.1.9.4 (Faul et al., 2007).

## Results

### Minigene Analyses of the Effects of SLC22A1 rs35854239 on Splicing

We used minigene assays to quantify the percentage of alternatively spliced transcripts in the 8 bp duplication allele of the rs35854239 variant. For this purpose, exon 7, including 306 bp upstream and 310 bp downstream of the flanking intronic regions, was cloned in the minigene vector pSPL3b between the exons 1 and 2 of the rabbit β-globulin gene. Next to the construct carrying the 8 bp duplication allele, the wild-type allele was also cloned and used as a control in the analyses (Figure 1B). Two independent minigene clones containing the duplication allele were analyzed to account for potential artifacts from the quality of the clones and the DNA preparation. The minigene constructs were transiently transfected into HepG2 and Huh7 cells, and the resulting correctly and alternatively spliced transcripts were quantified 48 hours later using three independent quantification techniques: semi-quantitative PCR, pyrosequencing and deep RNA-sequencing. In all cases, first, total RNA was reverse transcribed into cDNA. For the semi-quantitative PCR, the spliced SLC22A1 exon 7 was amplified by PCR using primers within the flanking exons of the rabbit β-globulin gene. The PCR products were separated by gel electrophoresis to enable the selective identification of the correctly and the alternatively spliced transcripts, and the band intensities were quantified (Figure 1C). As expected, the wild-type allele was spliced 100% correctly. However, the duplication allele was only spliced 47% correctly (range 42–51%) in transiently transfected Huh7 cells and 52% correctly (range 46–58%) in HepG2 cells (data not shown).

Next, we used pyrosequencing to quantify more precisely the ratio of the alternatively spliced transcripts. The pyrosequencing quantification method was validated by calibration series of vectors encoding the correctly or alternatively spliced minigene (Supplementary Figure S1). The pyrosequencing-based quantification showed that in Huh7 cells, the duplication allele was correctly spliced in 49% (range 46–52%) of all transcripts (Figure 1D). This was highly comparable with the previously semi-quantitatively determined ratios. In HepG2 cells, the duplication allele was correctly spliced in 57% (range 55–60%) of all transcripts.

Finally, the minigene insertion allele clone 1 spliced in Huh7 or HepG2 cells at 48 h was reanalyzed using deep massive-parallel sequencing (Figure 1E). The average depth of targeted RNA sequencing was 59,135 reads (range 32,902–84,691). The quantification of reads carrying the 8 bp insertion as a result of alternative splicing showed a percentage of 62% correctly spliced minigene in both cell lines. These results confirm the alternative splicing of rs35854239. More importantly, these results suggest that the 8 bp duplication causes erroneous splicing in only a part of the transcripts. Estimated by the data of all experiments maximally 52% of the transcripts are erroneously spliced. Thus, our *in vitro* data suggest that even in homozygous carriers of the duplication about the half of OCT1 transcripts will be correctly spliced.

### Effects of SLC22A1 Exon 7 Genetic Variants on rs35854239 Splicing

**FIGURE 2:**
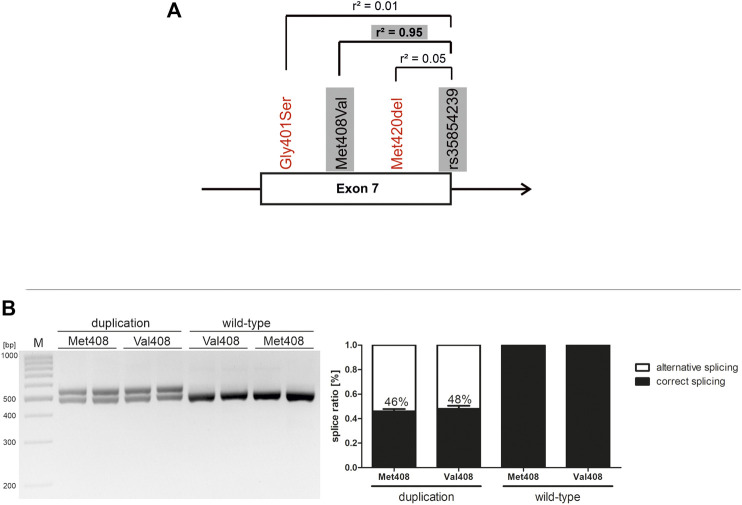
Effects of Met408Val on rs35854239 and on exon 7 splicing **(A)** Localization of rs35854239 at the exon 7-intron 7 junction of the SLC22A1 gene. Known variants reducing OCT1 function in exon 7 of the SLC22A1 gene are shown in red. The highly genetically linked variants that are separately analyzed here are highlighted in gray **(B)** The Met408Val polymorphism was introduced by site-directed mutagenesis into both minigenes: harboring the wild-type and the duplication alleles of rs35854239. After transient transfection in Huh7 cells, spliced transcripts were amplified and separated by gel electrophoresis and afterwards, band intensities of correctly and alternatively spliced transcripts were quantified. Percentages within boxes represent relative values of correctly spliced minigene. Shown are means and standard errors of the means of three independent experiments.

Within the SLC22A1 gene, exon 7 harbors the highest density of coding functionally relevant polymorphisms. Thereof, the Met408Val substitution is almost completely linked to the rs35854239 duplication (Figure 2A). Under native conditions, it could not be excluded that the coding variant substantially contributes to the effects of splicing. Here were took advantage of the minigene technique and addressed separately the effects of the two variants on splicing. The Val408Met substitution alone did not significantly affect splicing, neither on duplication nor on wild-type rs35854239 background (Figure 2B). Therefore, it could be concluded that the effects on splicing are completely caused by the rs35854239 duplication and there is no contribution of the highly linked Met408Val.

### Functional Characterization of the Protein Encoded by the Alternatively Spliced Transcript

**FIGURE 3:**
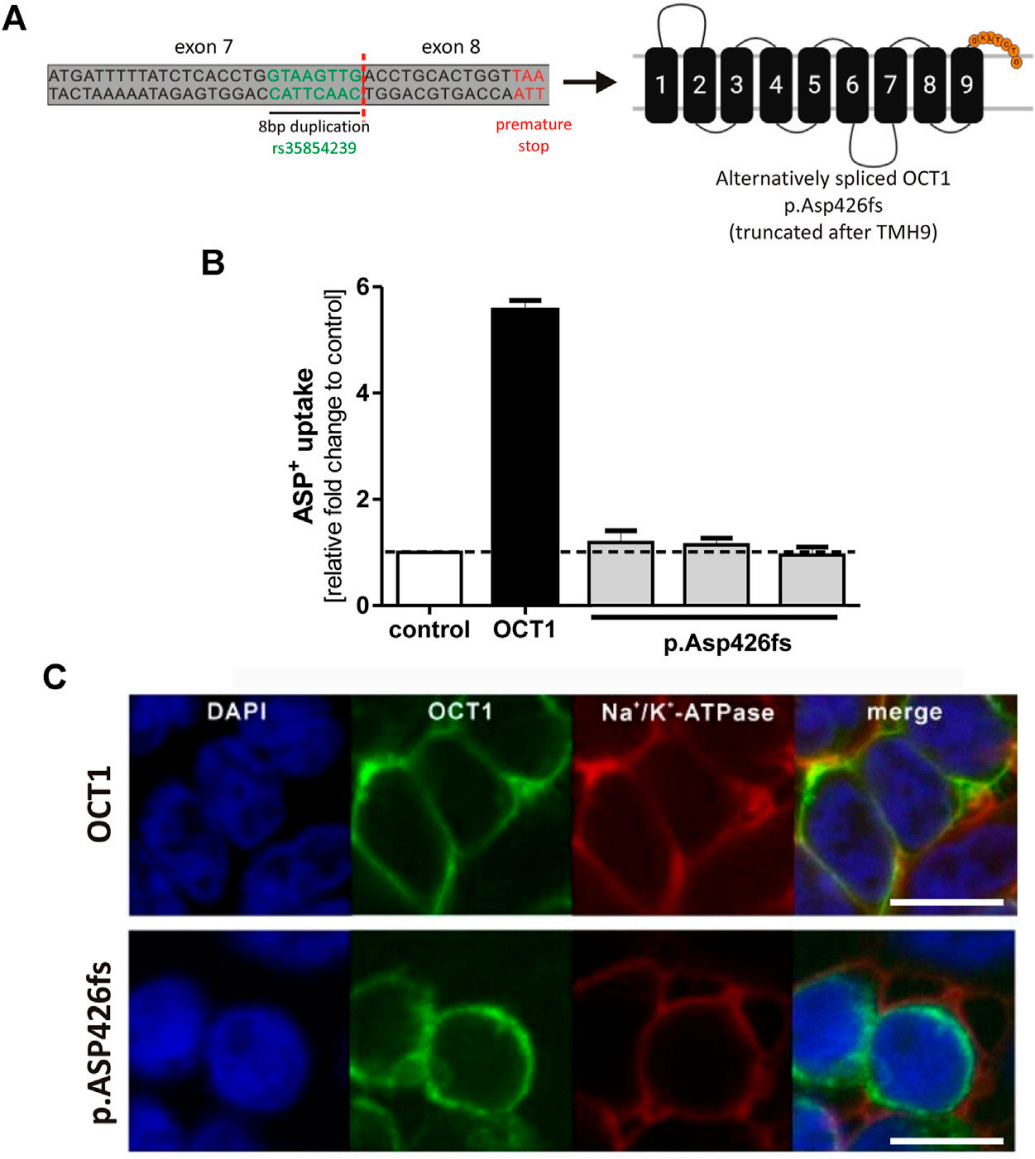
Effects of the alternatively spliced OCT1 protein p.Asp426fs on OCT1 function **(A)** Alternative splicing of rs35854239 leads to a premature stop after Asn431, resulting in an OCT1 protein that is truncated after transmembrane helix 9. **(B)** HEK293 cells transiently transfected with a vector coding for the alternatively spliced OCT1 p. Asp426fs were incubated for 2 min with 20 μM ASP^+^. The OCT1-mediated uptake was calculated as fold change compared to control cells (transfected with the empty vector). Transfection of wild-type OCT1 served as a positive control for functional transporter. Data are shown as mean and standard error of the mean (SEM) of three independent experiments. **(C)** Membrane localization was analyzed using immunofluorescence staining and confocal microscopy (magnification factor 63). The OCT1 antibody used for this purpose recognizes the intracellular loop of the protein between TMH6 and TMH7. OCT1 (green) was co-stained with Na^+^/K^+^-ATPase (red) as membrane marker. Scale bar indicates 10 µm. TMH, transmembrane helix.

The alternative splicing leads to an 8 bp longer exon 7, entailing a frame shift. This results in an altered amino acid sequence after codon 425 followed by a premature stop after seven amino acids. The resulting truncated OCT1 protein p. Asp426fs consists of the first nine TMHs only (Figure 3A).

To analyze whether the truncated OCT1 protein p. Asp426fs is able to function as an uptake transporter, the 8 bp insertion sequence was introduced between exon 7 and exon 8 of the OCT1 carrying pcDNA5 vector using site-directed mutagenesis. The resulting vector was transiently transfected into HEK293 cells and the uptake of the model OCT1 substrate ASP^+^ was compared to the uptake of the wild-type OCT1. Three independent clones of p.Asp426fs were analyzed. The alternatively spliced OCT1 protein showed no transport activity (Figure 3B). ASP^+^ uptake of all alternatively spliced OCT1 clones was at same levels as the empty pcDNA5 vector, indicating no OCT1-mediated substrate uptake.

Immunofluorescence staining revealed aberrant membrane localization of the alternatively spliced OCT1 (Figure 3C). This demonstrates that the truncated OCT1 protein, which results from alternative splicing of exon 7, is completely inactive and lacks correct membrane localization.

### Effects of rs35854239 on Splicing in Human Liver Samples

**FIGURE 4:**
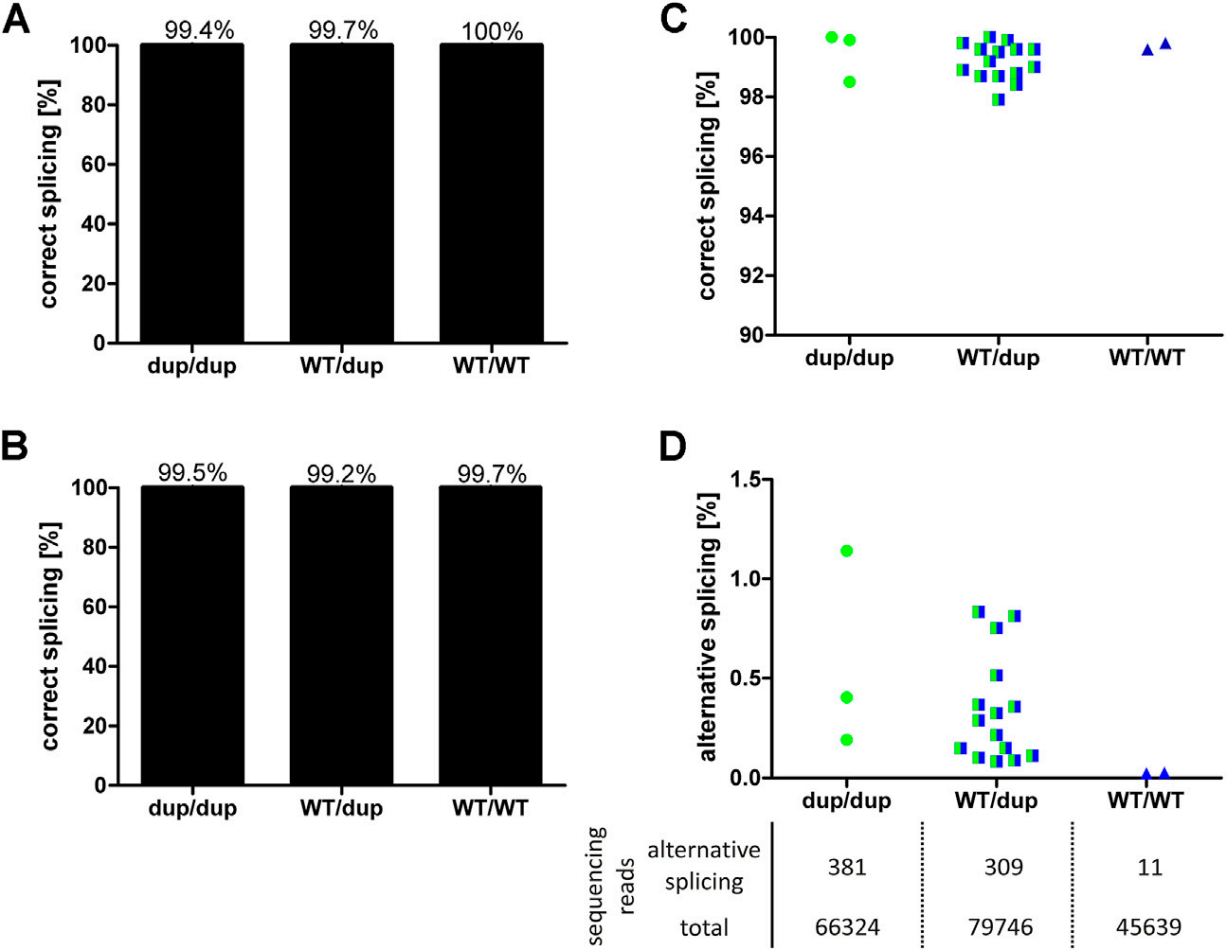
Effects of rs35854239 on OCT1 splicing in human liver samples. Splicing of the exon7-intron7 junction of OCT1 mRNA, depending on rs35854239 genotype was quantified using **(A)** pyrosequencing and **(B)** deep RNA-sequencing. On the left, the mean percentage of correctly spliced transcripts depending on the rs35854239 genotype is shown. The percentage of **(C)** correctly and **(D)** alternatively spliced transcripts for each individual sample is shown. **(D)** The mean sequencing depth for alternatively spliced and total transcripts is stated below. Dup, rs35854239 duplication allele; WT, wild-type SLC22A1 allele. Dup/dup: *n* = 3, WT/dup: *n* = 15, WT/WT: *n* = 2.

Minigene analyses in cell lines with hepatic origin showed that the 8 bp duplication leads to maximally 52% of alternatively spliced transcripts that encode a non-functional protein. In order to validate these results *in vivo*, we quantified the effects of the 8 bp duplication on OCT1 mRNA splicing in human liver. To this end, DNA from 24 liver samples was genotyped for rs35854239 and the correct splicing of the exon 7-intron 7 junction was quantified using pyrosequencing and deep RNA-sequencing (Figure 4).

Using pyrosequencing, we observed 99% correctly spliced SLC22A1 mRNA irrespective of the genotype of the liver donors. Alternative splicing of OCT1 in liver samples from homozygous or heterozygous 8 bp duplication allele carriers appeared with a maximum of 2.1% (Figure 4A). This percentage is far below the observed results in minigene experiments (Figure 1) but is still substantially higher than the observed 0.4% in the SLC22A1 mRNA from donors with wild-type genotype, which can only be spliced correctly.

Using deep RNA-sequencing, we detected very low levels of alternatively spliced transcripts that were, however, dependent on the rs35854239 genotype (Figure 4B). The average depth of sequencing was 74,326 reads per RNA sample (range 41,116–133,715). The liver samples from homozygous duplication allele carriers showed mean values of 0.58% alternatively spliced transcripts (range 0.19–1.14%). In heterozygous genotypes, alternative splicing was detected with a mean of 0.36% (range 0.08–0.83%). The samples of the homozygous wild-type allele carriers showed a mean of 0.02% alternatively spliced transcripts indicating very low levels of possible contamination with this highly sensitive method. In conclusion, both techniques demonstrated that despite the close to 50% probability of alternative splicing of the rs35854239 duplication allele estimated by the minigene assays, alternatively spliced OCT1 could only barely be detected in human liver samples. These results suggest that the alternatively spliced transcripts may be recognized and rapidly degraded under native conditions.

**FIGURE 5:**
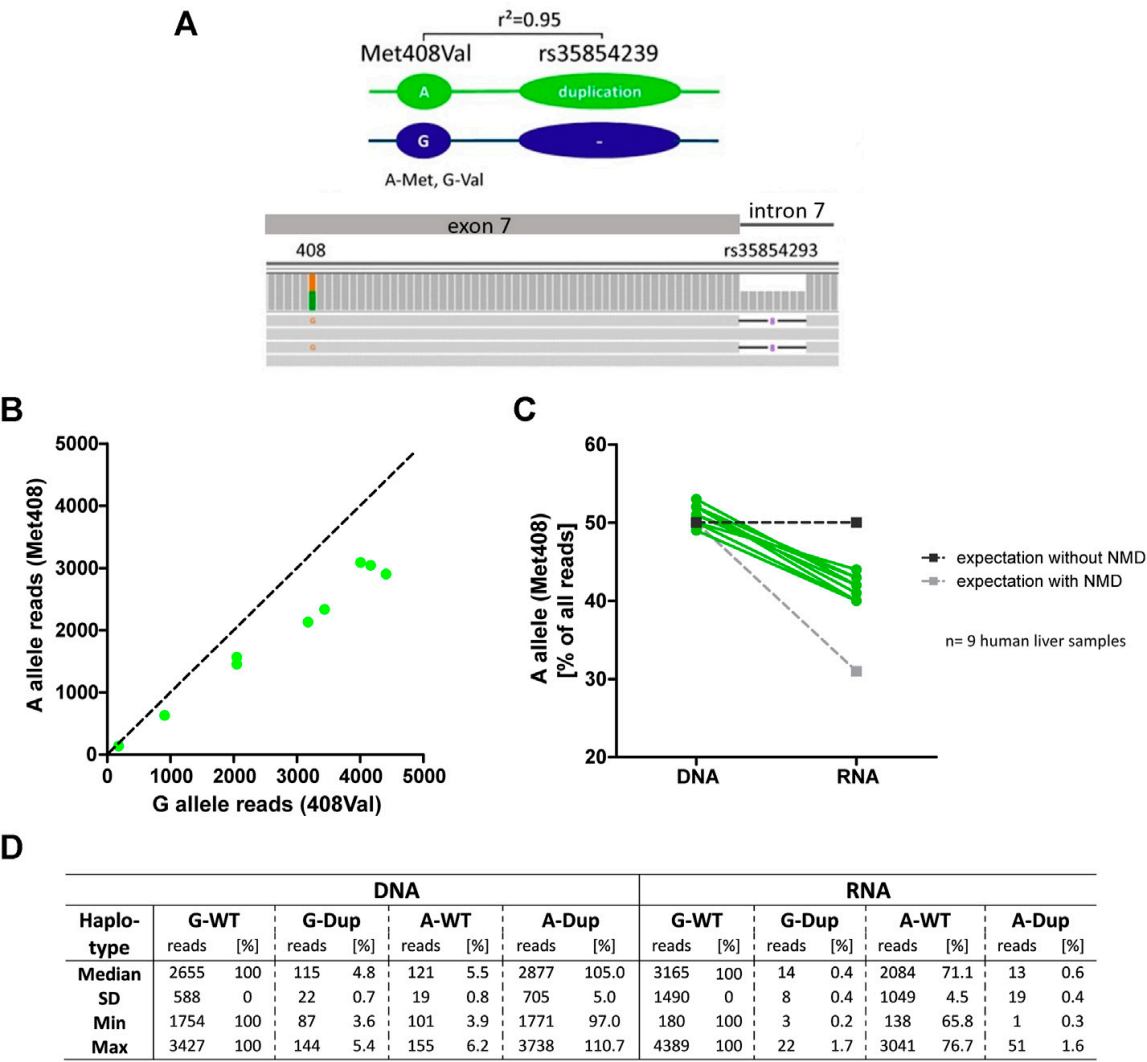
Allelic expression imbalance in heterozygous carriers of rs35854239 **(A)** The duplication allele of rs35854239 is highly linked to the A-allele of Met408. The abundance of both Met408Val alleles was analyzed on DNA and RNA level in heterozygous Met408Val/rs35854239 human liver samples using deep RNA-sequencing. **(B)** The abundance of both Met408Val alleles on RNA level was analyzed and **(C)** the percentage of the A-allele (Met408) on DNA and RNA levels was compared in nine human liver samples (green). Dashed lines represent expected allele balance without (dark gray) and with (light gray) non sense-mediated mRNA decay (NMD). **(D)** Sequencing depth is given in absolute reads and relative to the Val408 G-allele and rs35854239 wild-type haplotype.

To verify this, we performed an allelic expression imbalance analysis in the human liver samples. We took advantage of the strong genetic linkage between the duplication allele of rs35854239 and the A-allele of the coding variant Met408Val (rs628031, 1222A>G, *r* ^2^ = 0.95; Figure 5A). Based on the strong linkage, in heterozygous carriers of the rs35854239 duplication and Met408Val A-allele haplotype, a degradation of alternatively spliced OCT1 could be detected as a lower abundance of the Met408 A-allele in the RNA transcripts compared to the expected 50% of the DNA reads (Figure 5B). We used all nine liver samples from which both DNA and RNA was available and applied deep sequencing for quantification. While the A-allele was detected in 50% of the DNA reads (range 49–53%), the abundance in RNA was significantly decreased to 42% (range 40–44%, *p* = 2.77 × 10^−7^, paired *t*-test; Figure 5C). This result supports the degradation of the alternatively spliced transcripts and suggests that the presence of the rs35854239 duplication will result in the reduction of OCT1 mRNA levels, and as a consequence OCT1 protein in general.

In addition, more precise analyses of the sequencing reads suggest that the correct “canonical” splicing may be preferred under native conditions. Indeed, reads of alternatively spliced RNA carrying the A-allele of Met408Val were almost not detectable (Figure 5D). However, the reads of correctly spliced RNA carrying A-allele Met408Val were 71.3% of the G-allele reads (range from 65.8 to 76.7%) instead of the expected 50% from the minigene analyses. This suggests that in parallel to degradation of the alternatively spliced transcripts also a preference for correct splicing of rs35854239 duplication allele under native conditions may exist.

**FIGURE 6:**
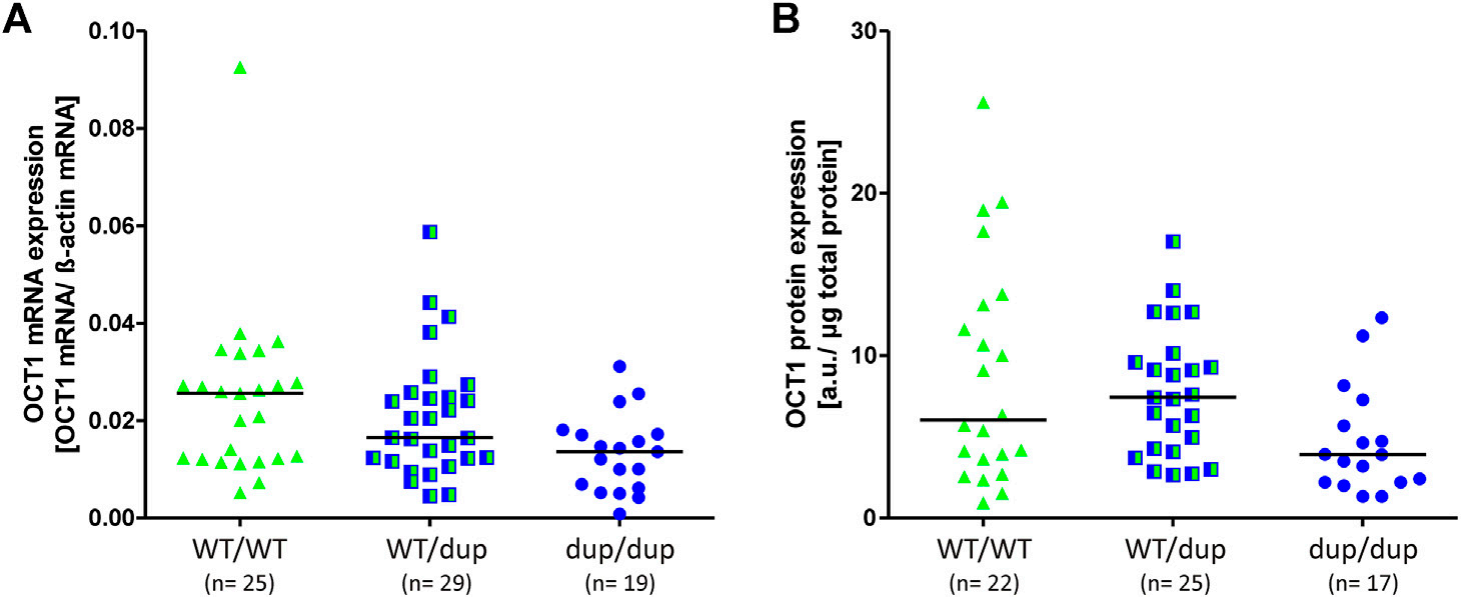
Effect of rs35854239 on OCT1 **(A)** mRNA and **(B)** protein expression. Median OCT1 **(A)** mRNA expression and **(B)** protein expression was analyzed in 73 liver samples depending on the rs35854239 genotype. Dup, rs35854239 duplication allele; WT, wild-type OCT1 allele; a. u., arbitrary unit.

To address this, we analyzed total OCT1 mRNA and protein expression in 73 human liver samples. The liver samples had been characterized for their OCT1 expression before (Nies et al., 2009). We included only those samples lacking the Arg61Cys substitution, which is known to significantly affect OCT1 protein levels in the liver (Supplementary table S2 and (Nies et al., 2009) by affecting the correct membrane localization (Seitz et al., 2015). The OCT1 mRNA expression was on median 47% lower in homozygous duplication than in homozygous wild-type rs35854239 allele carriers (median of 0.014 and 0.026 transcripts per beta-actin transcript, respectively; *p* = 0.007; Figure 6A). The OCT1 protein levels were on median 35% lower in homozygous duplication than in homozygous wild-type rs35854239 allele carriers (median of 3.91 and 6.04, respectively; *p* = 0.045; Figure 6B). However, although statistically significant, the effects of rs35854239 genotypes could explain only 9% of the variability of mRNA and protein expression in this sample set.

### Effects of rs35854239 Duplication on the Pharmacokinetics of Sumatriptan and Fenoterol

**FIGURE 7:**
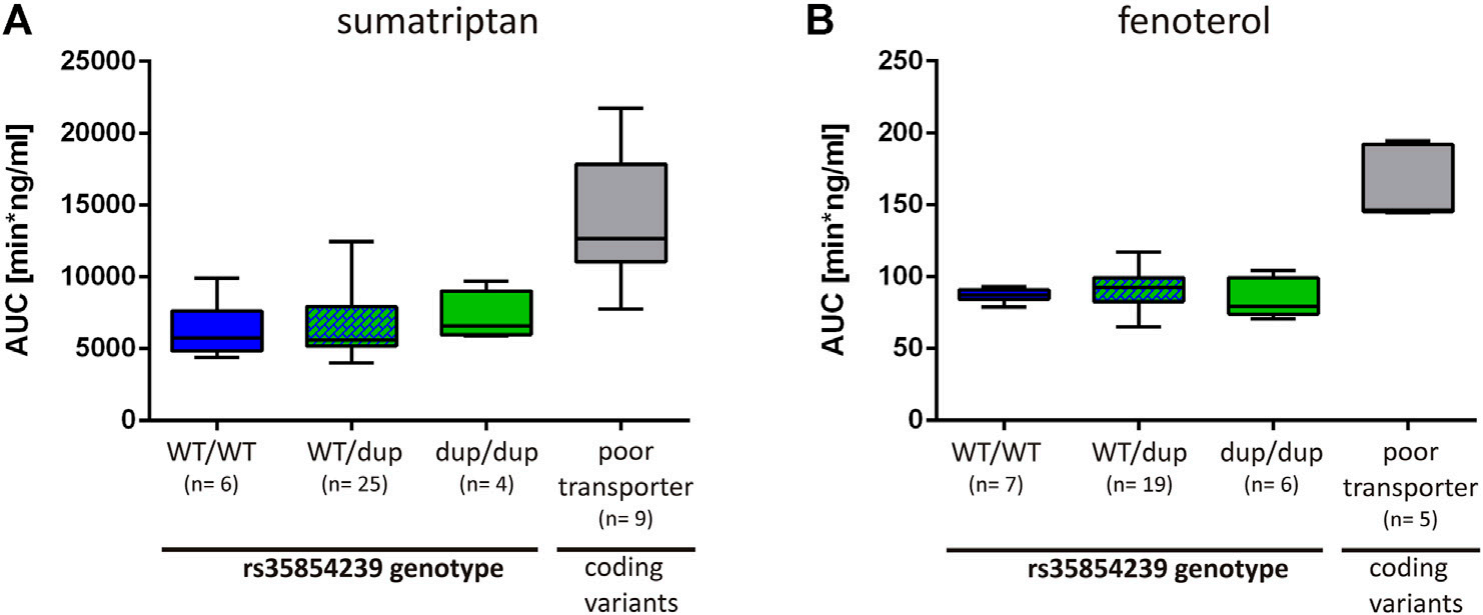
Effects of rs35854239 on the pharmacokinetics of **(A)** sumatriptan and **(B)** fenoterol. The AUC of **(A)** sumatriptan and **(B)** fenoterol is depicted depending on the rs35854239 genotype and compared to the OCT1 phenotype (poor transporters). Poor transporters comprise homozygous or compound heterozygous carriers of the OCT1 alleles *3, *4, and *5 harboring the coding variants Arg61Cys, Gly401Ser or Gly465Arg, respectively. Dup, rs35854239 duplication allele; WT, wild-type OCT1 allele; AUC, area under the plasma concentration-time curve. Boxplots show median, lower (25%) and upper (75%) quartiles.

Finally, we analyzed to what extend the decrease of OCT1 expression in carriers of the rs35854239 duplication allele leads to changes in sumatriptan and fenoterol pharmacokinetics in humans. We took advantage of the existing studies on the effects of OCT1 genotypes on the pharmacokinetics of both drugs (Matthaei et al., 2016; Tzvetkov et al., 2018) and analyzed them in the context of the rs35854239 genotype. The AUC of sumatriptan was slightly increased in homozygous rs35854239 duplication allele carriers compared to the wild-type (means of 7187 vs. 6277 min × ng/ ml, respectively, Figure 7A). However, this increase was not significant and was on average by 14% compared to the observed 127% increase in poor OCT1 transporters (homozygous or compound heterozygous carriers of the coding variants Arg61Cys, Gly401Ser, Gly465Arg) observed in the same study.

Even more, the AUC of fenoterol was not higher in homozygous carriers of the rs35854239 duplication allele compared to the wild-type (means of 84.25 vs. 86.84 min × ng/ml, respectively; Figure 7B). In comparison, poor OCT1 transporters showed 1.89-fold higher AUCs for fenoterol. This data suggests that compared to the well-known loss-of-function coding variants, the 8 bp duplication shows only limited effects on drugs pharmacokinetics.

## Discussion

The eight base pairs duplication at the exon 7-intron 7 junction (rs35854239) has been previously suggested to cause erroneous splicing of OCT1 by introducing an alternative splice site in the intronic sequence of intron 7. In this study, we confirm the alternative splicing and give more precise quantitative information about the effects on OCT1 expression and activity in order to better estimate the contribution of this variant to the highly inter-individual variability in OCT1 activity.

This study built up on the previous findings of Kim et al. (Kim et al., 2017). We confirmed the findings of Kim et al. that the 8 bp duplication causes alternative splicing. We did this both by using minigene assays (Figure 1) and by detecting (a low level) of the alternatively spliced transcript in human liver samples (Figure 4). We also confirmed the finding of Kim et al. that the alternatively spliced transcript is not leading to a functional protein (Figure 2).

The major contribution of this study beyond the previously known is the precise quantification of the effects of the 8 bp duplication rs35854239. We used minigene analyses to quantify the effects on splicing (Figure 1) and to confirm that these effects are caused by the 8 bp duplication and not by the highly genetically linked variant Met408Val. We quantified the effects of the 8 bp duplication on total OCT1 expression in human liver both on mRNA and on protein levels (Figure 6) and finally we analyzed the association of the splice variants with the pharmacokinetics of drugs that are well known OCT1 substrates (Figure 7). This will enable us to better evaluate the contribution of the rs35854239 duplication to the high genetic and thus to the high functional variability of OCT1 in humans.

Our data suggest that the 8 bp duplication allele can cause erroneous splicing of up to 50% of the transcripts, but, probably due to mRNA decay, the number of detectable erroneously spliced transcripts in the human liver is very low. Thus, homozygous carriers of the duplication allele are characterized by decreased expression of the correctly spliced transcripts resulting in a median decrease of OCT1 protein expression by 35% in the human liver. However, the rs35854239 effects explained only 9% of the highly variable SLC22A1 mRNA expression in humans (Figure 6), and the 8 bp duplication was not associated with significant changes in the pharmacokinetics of known OCT1 substrates, i.e. sumatriptan and fenoterol (Figure 7).

The rs35854239 ins/del variant results in duplication of the originally existing donor splice site of intron 7, giving a possibility of alternative, but also keeping the possibility of correct “canonical” splicing. Here, we demonstrated that both donor splice sites are operative. Depending on the cellular system and the quantification technique used, intensive processing of both splice sites with a slight preference for the usage of the original “correct” donor splice site was suggested (Figure 1).

These numbers are in contrast to the almost undetectable alternatively spliced transcripts in the human liver. These discrepancies could be explained by a nonsense-mediated mRNA decay (NMD) as suggested previously by Kim et al. (Kim et al., 2017). Here, we provide multiple confirmations for an mRNA decay of the incorrectly spliced OCT1 transcripts. Firstly, using minigene constructs that use rabbit flanking exons, and thus are not affected by NMD, we observed a substantially higher percentage of the alternatively spliced transcripts. Secondly, we applied allelic expression imbalance analyses, taking advantage of the genetically highly linked coding variant Met408Val, and were able to demonstrate imbalance in the expression of the duplication-linked allele Met408 (Figure 5). Finally, we demonstrated significantly reduced expression of the correctly spliced transcripts in homozygous carriers of the duplication allele (Figure 6).

Even if not degraded, the alternatively spliced transcript encodes a truncated protein p.Asp426fs. This protein is missing TMHs 10 to 12, which are essential for OCT1 activity (Shu et al., 2003; Gorboulev et al., 2005; Egenberger et al., 2012) and is not localized correctly at in the plasma membrane (Figure 3). Therefore, only the correctly spliced transcripts can contribute to OCT1 activity.

A major hypothesis of this study was that the 8 bp duplication that affects splicing may explain a major part of OCT1 expression variability. OCT1 mRNA expression in the human liver varied more than 100-fold between individuals (Nies et al., 2009; O'Brien et al., 2013). In the 73 liver samples analyzed in this study, we observed 23-fold variability in OCT1 expression. Despite a median reduction of OCT1 expression by almost 50% in homozygous duplication carriers, the genetic variant could explain less than 10% of the general variability. Taken together with the lack of strong effects of genetic variants in the SLC22A1 promoter (Bokelmann et al., 2018), only minor effects of *cis*-acting variants could be concluded. Systematic analyses of the genetic component in the variability of OCT1 expression are highly complicated, as multiple sampling of the same individuals or sampling within multiple members of the family are required. However, there are already some data suggesting that *trans*-acting variants or non-genetic factors may play a role. Indeed, genetic variants in transcription factors known to regulate OCT1 expression were associated with OCT1 expression (O’Brien et al., 2013), and disease conditions such as cholestasis may play a role (Nies et al., 2009). There are a number of other transcriptional factors suggested to regulate OCT1 expression, e.g. CCAAT/enhancer binding proteins, pregnane X receptor (PXR), farnesoid X receptor (FXR), and glucocorticoid receptor (GR)(Saborowski et al., 2006; Rulcova et al., 2013; Hyrsova et al., 2016). It will be interesting to study whether genetic variants within them may have *cis*-effects on OCT1 expression.

Regarding potential effects of rs35854239 on pharmacokinetics, our data suggest that the impact on OCT1 expression caused by the duplication does not significantly affect the pharmacokinetics of known OCT1 substrates in humans. We were not able to detect significant differences in the AUCs of sumatriptan or fenoterol depending on the presence or absence of the rs35854239 duplication, in contrast to the clear and highly significant effects of the well-known coding OCT1 variants (Figure 7). Indeed, these two studies were not designed to address effects of rs35854239. However, based on the high frequency of this variant, we had a power of 80% to detect the increase in AUC of 47% and higher for sumatriptan, and of 15% or higher for fenoterol. The significant, but less prominent effects of this variant are in line with previous reports (Kim et al., 2017).

One explanation may be that the 50% reduction of OCT1 expression is not sufficient to affect substantially the pharmacokinetics of substrates in humans. Our previous studies on the effects of coding genetic variants on OCT1 activity clearly demonstrated that reduction of the typical OCT1 activity by more than 50% (OCT1 gene dose of less than 1 with a typical gene dose of 2) is necessary to cause measurable changes in drug pharmacokinetics (Tzvetkov et al., 2018; Matthaei et al., 2019). This is in line with the lack of evidence for an independent association of rs35854239 with serum isobutyrylcarnitine levels in the Kim et al. study (Kim et al., 2017). The authors of this study also interpreted this as a consequence of the small effect size of the duplication.

Multiple studies reported an association of the Met408Val variant with pharmacokinetics or efficacy of drugs that are potential OCT1 substrates (White et al., 2006; Shikata et al., 2007; Wang et al., 2008; Kim et al., 2009; Takahashi et al., 2010; Tarasova et al., 2012; Koren-Michowitz et al., 2014; Vaidya et al., 2015). However, those associations were difficult to interpret, as no significant effect of this coding variant on transport activity could be demonstrated (Shu et al., 2003; Shu et al., 2007; White et al., 2010; Nies et al., 2014; Tzvetkov et al., 2014; Seitz et al., 2015). The rs35854239 variant is almost completely genetically linked to Met408Val (*r* ^2^ of 0.95). Our data strongly suggest the rs35854239 variant as causative variant due to its effects on splicing and thus on OCT1 expression. However, we also demonstrated that these effects are less prominent than the influences of other common coding variants of the gene, which lead to reduced or loss of OCT1 function (Arg61Cys, Cys88Arg, Gly401Ser and Gly465Arg). Therefore, the observed strong association with Met408Val or directly with rs35854239, but absence of association with the highly functional amino acid substitutions listed above are still difficult to explain.

In conclusion, using minigene analyses we were able to quantify that the common naturally occurring 8 bp duplication at the exon 7-intron 7 junction (rs35854239) causes alternative splicing in approximately 50% of the cases. The alternatively spliced transcripts are degraded under native conditions in the liver, but even if stable they are not able to encode the active protein (as demonstrated here using expression in HEK293 cells), and thus result in a significant decrease in OCT1 expression detectable both on mRNA and protein levels. However, although very common (minor allele frequency of 40.6%), the decrease in expression, taken together with the high general variability of OCT1 expression, was not sufficient to cause strong effects on drug pharmacokinetics (as demonstrated by analyzing the effects of the variant on pharmacokinetics of sumatriptan and fenoterol in healthy individuals).

## Data Availability

The original contributions presented in the study are publicly available. This data can be found here: NCBI repository, accession number: PRJNA720275 (https://www.ncbi.nlm.nih.gov/sra/PRJNA720275)

## References

[B1] Arimany-Nardi C. Minuesa G. Keller T. Erkizia I. Koepsell H. Martinez-Picado J. (2016). Role of Human Organic Cation Transporter 1 (hOCT1) Polymorphisms in Lamivudine (3TC) Uptake and Drug-Drug Interactions. Front. Pharmacol. 7, 175. 10.3389/fphar.2016.00175 27445813 PMC4919327

[B2] Becker M. L. Visser L. E. van Schaik R. H. N. Hofman A. Uitterlinden A. G. Stricker B. H. C. (2011). OCT1 Polymorphism Is Associated with Response and Survival Time in Anti-parkinsonian Drug Users. Neurogenetics 12 (1), 79–82. 10.1007/s10048-010-0254-5 20680652 PMC3029819

[B3] Bokelmann K. Brockmöller J. Tzvetkov M. (2018). Impact of Promoter Polymorphisms on the Transcriptional Regulation of the Organic Cation Transporter OCT1 (SLC22A1). J. Pers Med. 8 (4), 42. 10.3390/jpm8040042 PMC6313513 30544975

[B4] Chen L. Shu Y. Liang X. Chen E. C. Yee S. W. Zur A. A. (2014). OCT1 Is a High-Capacity Thiamine Transporter that Regulates Hepatic Steatosis and Is a Target of Metformin. Proc. Natl. Acad. Sci. USA 111 (27), 9983–9988. 10.1073/pnas.1314939111 24961373 PMC4103324

[B5] Drozdzik M. Busch D. Lapczuk J. Müller J. Ostrowski M. Kurzawski M. (2019). Protein Abundance of Clinically Relevant Drug Transporters in the Human Liver and Intestine: A Comparative Analysis in Paired Tissue Specimens. Clin. Pharmacol. Ther. 105 (5), 1204–1212. 10.1002/cpt.1301 30447067

[B6] Egenberger B. Gorboulev V. Keller T. Gorbunov D. Gottlieb N. Geiger D. (2012). A Substrate Binding Hinge Domain Is Critical for Transport-Related Structural Changes of Organic Cation Transporter 1*. J. Biol. Chem. 287 (37), 31561–31573. 10.1074/jbc.M112.388793 22810231 PMC3438988

[B7] Faul F. Erdfelder E. Lang A.-G. Buchner A. (2007). G*Power 3: a Flexible Statistical Power Analysis Program for the Social, Behavioral, and Biomedical Sciences. Behav. Res. Methods 39 (2), 175–191. 10.3758/BF03193146 17695343

[B8] Fukuda T. Chidambaran V. Mizuno T. Venkatasubramanian R. Ngamprasertwong P. Olbrecht V. (2013). OCT1genetic Variants Influence the Pharmacokinetics of Morphine in Children. Pharmacogenomics 14 (10), 1141–1151. 10.2217/pgs.13.94 23859569 PMC4116670

[B9] Gorboulev V. Shatskaya N. Volk C. Koepsell H. (2005). Subtype-specific Affinity for Corticosterone of Rat Organic Cation Transporters rOCT1 and rOCT2 Depends on Three Amino Acids within the Substrate Binding Region. Mol. Pharmacol. 67 (5), 1612–1619. 10.1124/mol.104.008821 15662045

[B10] Grinfeld J. Gerrard G. Alikian M. Alonso-Dominguez J. Ale S. Valgañon M. (2013). A Common Novel Splice Variant ofSLC22A1(OCT1)is Associated with Impaired Responses to Imatinib in Patients with Chronic Myeloid Leukaemia. Br. J. Haematol. 163 (5), 631–639. 10.1111/bjh.12591 24117365

[B11] Hyrsova L. Smutny T. Carazo A. Moravcik S. Mandikova J. Trejtnar F. (2016). The Pregnane X Receptor Down-Regulates Organic Cation Transporter 1 (SLC22A1) in Human Hepatocytes by Competing for (“squelching”) SRC-1 Coactivator. Br. J. Pharmacol. 173 (10), 1703–1715. 10.1111/bph.13472 26920453 PMC4842917

[B12] Kerb R. Brinkmann U. Chatskaia N. Gorbunov D. Gorboulev V. Mornhinweg E. (2002). Identification of Genetic Variations of the Human Organic Cation Transporter hOCT1 and Their Functional Consequences. Pharmacogenetics 12 (8), 591–595. 10.1097/00008571-200211000-00002 12439218

[B13] Kim D. H. Sriharsha L. Xu W. Kamel-Reid S. Liu X. Siminovitch K. (2009). Clinical Relevance of a Pharmacogenetic Approach Using Multiple Candidate Genes to Predict Response and Resistance to Imatinib Therapy in Chronic Myeloid Leukemia. Clin. Cancer Res. 15 (14), 4750–4758. 10.1158/1078-0432.CCR-09-0145 19584153

[B14] Kim H. I. Raffler J. Lu W. Lee J.-J. Abbey D. Saleheen D. (2017). Fine Mapping and Functional Analysis Reveal a Role of SLC22A1 in Acylcarnitine Transport. Am. J. Hum. Genet. 101 (4), 489–502. 10.1016/j.ajhg.2017.08.008 28942964 PMC5630162

[B15] Koehler M. R. Wissinger B. Gorboulev V. Koepsell H. Schmid M. (1997). The Two Human Organic Cation Transporter Genes SLC22A1 and SLC22A2 Are Located on Chromosome 6q26. Cytogenet. Cel Genet 79 (3-4), 198–200. 10.1159/000134720 9605850

[B16] Koren-Michowitz M. Buzaglo Z. Ribakovsky E. Schwarz M. Pessach I. Shimoni A. (2014). OCT1 Genetic Variants Are Associated with Long Term Outcomes in Imatinib Treated Chronic Myeloid Leukemia Patients. Eur. J. Haematol. 92 (4), 283–288. 10.1111/ejh.12235 24215657

[B49] Langmead B. Salzberg S. L. (2012). Fast gapped-read alignment with Bowtie 2. Nature meth. 9, 357–359. 10.1038/nmeth.1923 PMC3322381 22388286

[B17] Matthaei J. Kuron D. Faltraco F. Knoch T. Dos Santos Pereira J. Abu Abed M. (2016). OCT1 Mediates Hepatic Uptake of Sumatriptan and Loss-Of-functionOCT1polymorphisms Affect Sumatriptan Pharmacokinetics. Clin. Pharmacol. Ther. 99 (6), 633–641. 10.1002/cpt.317 26659468

[B50] Matthaei J. Seitz T. Jensen O. Tann A. Prukop T. Tadjerpisheh M. V. (2019). OCT1 Deficiency Affects Hepatocellular Concentrations and Pharmacokinetics of Cycloguanil, the Active Metabolite of the Antimalarial Drug Proguanil. Clin. Pharmacol. Ther. 105, 190–200. 10.1002/cpt.1128 29882324

[B18] Morais S. M. d. Wilkinson G. R. Blaisdell J. Nakamura K. Meyer U. A. Goldstein J. A. (1994). The Major Genetic Defect Responsible for the Polymorphism of S-Mephenytoin Metabolism in Humans. J. Biol. Chem. 269 (22), 15419–15422. 10.1016/S0021-9258(17)40694-6 8195181

[B19] Nies A. T. Koepsell H. Winter S. Burk O. Klein K. Kerb R. (2009). Expression of Organic Cation Transporters OCT1 (SLC22A1) and OCT3 (SLC22A3) Is Affected by Genetic Factors and Cholestasis in Human Liver. Hepatology 50 (4), 1227–1240. 10.1002/hep.23103 19591196

[B20] Nies A. T. Niemi M. Burk O. Winter S. Zanger U. M. Stieger B. (2013). Genetics Is a Major Determinant of Expression of the Human Hepatic Uptake Transporter OATP1B1, but Not of OATP1B3 and OATP2B1. Genome Med. 5 (1), 1. 10.1186/gm405 23311897 PMC3706890

[B21] Nies A. T. Schaeffeler E. van der Kuip H. Cascorbi I. Bruhn O. Kneba M. (2014). Cellular Uptake of Imatinib into Leukemic Cells Is Independent of Human Organic Cation Transporter 1 (OCT1). Clin. Cancer Res. 20 (4), 985–994. 10.1158/1078-0432.CCR-13-1999 24352644 PMC3932302

[B22] O’Brien V. P. Bokelmann K. Ramírez J. Jobst K. Ratain M. J. Brockmöller J. (2013). Hepatocyte Nuclear Factor 1 Regulates the Expression of the Organic Cation Transporter 1 via Binding to an Evolutionary Conserved Region in Intron 1 of the OCT1 Gene. J. Pharmacol. Exp. Ther. 347 (1), 181–192. 10.1124/jpet.113.206359 23922447 PMC3781413

[B23] Rulcova A. Krausova L. Smutny T. Vrzal R. Dvorak Z. Jover R. (2013). Glucocorticoid Receptor Regulates Organic Cation Transporter 1 (OCT1, SLC22A1) Expression via HNF4α Upregulation in Primary Human Hepatocytes. Pharmacol. Rep. 65 (5), 1322–1335. 10.1016/s1734-1140(13)71491-9 24399729

[B24] Saborowski M. Kullak-Ublick G. A. Eloranta J. J. (2006). The Human Organic Cation Transporter-1 Gene Is Transactivated by Hepatocyte Nuclear Factor-4α. J. Pharmacol. Exp. Ther. 317 (2), 778–785. 10.1124/jpet.105.099929 16436500

[B25] Schaeffeler E. Hellerbrand C. Nies A. T. Winter S. Kruck S. Hofmann U. (2011). DNA Methylation Is Associated with Downregulation of the Organic Cation Transporter OCT1 (SLC22A1) in Human Hepatocellular Carcinoma. Genome Med. 3 (12), 82. 10.1186/gm298 22196450 PMC3334547

[B26] Schaller L. Lauschke V. M. (2019). The Genetic Landscape of the Human Solute Carrier (SLC) Transporter Superfamily. Hum. Genet. 138 (11-12), 1359–1377. 10.1007/s00439-019-02081-x 31679053 PMC6874521

[B27] Seitz T. Stalmann R. Dalila N. Chen J. Pojar S. Dos Santos Pereira J. N. (2015). Global Genetic Analyses Reveal Strong Inter-ethnic Variability in the Loss of Activity of the Organic Cation Transporter OCT1. Genome Med. 7 (1), 56. 10.1186/s13073-015-0172-0 26157489 PMC4495841

[B28] Shikata E. Yamamoto R. Takane H. Shigemasa C. Ikeda T. Otsubo K. (2007). Human Organic Cation Transporter (OCT1 and OCT2) Gene Polymorphisms and Therapeutic Effects of Metformin. J. Hum. Genet. 52 (2), 117–122. 10.1007/s10038-006-0087-0 17111267

[B29] Shu Y. Brown C. Castro R. Shi R. Lin E. Owen R. (2008). Effect of Genetic Variation in the Organic Cation Transporter 1, OCT1, on Metformin Pharmacokinetics. Clin. Pharmacol. Ther. 83 (2), 273–280. 10.1038/sj.clpt.6100275 17609683 PMC2976713

[B30] Shu Y. Leabman M. K. Feng B. Mangravite L. M. Huang C. C. Stryke D. (2003). Evolutionary Conservation Predicts Function of Variants of the Human Organic Cation Transporter, OCT1. Proc. Natl. Acad. Sci. 100 (10), 5902–5907. 10.1073/pnas.0730858100 12719534 PMC156299

[B31] Shu Y. Sheardown S. A. Brown C. Owen R. P. Zhang S. Castro R. A. (2007). Effect of Genetic Variation in the Organic Cation Transporter 1 (OCT1) on Metformin Action. J. Clin. Invest. 117 (5), 1422–1431. 10.1172/JCI30558 17476361 PMC1857259

[B32] Smith P. K. Krohn R. I. Hermanson G. T. Mallia A. K. Gartner F. H. Provenzano M. D. (1985). Measurement of Protein Using Bicinchoninic Acid. Anal. Biochem. 150 (1), 76–85. 10.1016/0003-2697(85)90442-7 3843705

[B33] Stamer U. M. Musshoff F. Stüber F. Brockmöller J. Steffens M. Tzvetkov M. V. (2016). Loss-of-function Polymorphisms in the Organic Cation Transporter OCT1 Are Associated with Reduced Postoperative Tramadol Consumption. Pain 157 (11), 2467–2475. 10.1097/j.pain.0000000000000662 27541716

[B34] Takahashi N. Miura M. Scott S. A. Kagaya H. Kameoka Y. Tagawa H. (2010). Influence of CYP3A5 and Drug Transporter Polymorphisms on Imatinib Trough Concentration and Clinical Response Among Patients with Chronic Phase Chronic Myeloid Leukemia. J. Hum. Genet. 55 (11), 731–737. 10.1038/jhg.2010.98 20720558

[B35] Tarasova L. Kalnina I. Geldnere K. Bumbure A. Ritenberga R. Nikitina-Zake L. (2012). Association of Genetic Variation in the Organic Cation Transporters OCT1, OCT2 and Multidrug and Toxin Extrusion 1 Transporter Protein Genes with the Gastrointestinal Side Effects and Lower BMI in Metformin-Treated Type 2 Diabetes Patients. Pharmacogenet. Genom. 22 (9), 659–666. 10.1097/FPC.0b013e3283561666 22735389

[B36] Tzvetkov M. V. Dalila N. Faltraco F. (2016). “Genetic Variability in Organic Cation Transporters: Pathophysiological Manifestations and Consequences for Drug Pharmacokinetics and Efficacy,” in Organic Cation Transporters. Editors Ciarimboli G. Gautron S. Schlatter E. (Cham:Springer), 93–93.

[B37] Tzvetkov M. V. Dos Santos Pereira J. N. Meineke I. Saadatmand A. R. Stingl J. C. Brockmöller J. (2013). Morphine Is a Substrate of the Organic Cation Transporter OCT1 and Polymorphisms in OCT1 Gene Affect Morphine Pharmacokinetics after Codeine Administration. Biochem. Pharmacol. 86 (5), 666–678. 10.1016/j.bcp.2013.06.019 23835420

[B38] Tzvetkov M. V. Matthaei J. Pojar S. Faltraco F. Vogler S. Prukop T. (2018). Increased Systemic Exposure and Stronger Cardiovascular and Metabolic Adverse Reactions to Fenoterol in Individuals with HeritableOCT1Deficiency. Clin. Pharmacol. Ther. 103 (5), 868–878. 10.1002/cpt.812 28791698

[B39] Tzvetkov M. V. Saadatmand A. R. Bokelmann K. Meineke I. Kaiser R. Brockmöller J. (2012). Effects of OCT1 Polymorphisms on the Cellular Uptake, Plasma Concentrations and Efficacy of the 5-HT3 Antagonists Tropisetron and Ondansetron. Pharmacogenomics J. 12 (1), 22–29. 10.1038/tpj.2010.75 20921968

[B40] Tzvetkov M. V. Saadatmand A. R. Lötsch J. Tegeder I. Stingl J. C. Brockmöller J. (2011). Genetically Polymorphic OCT1: Another Piece in the Puzzle of the Variable Pharmacokinetics and Pharmacodynamics of the Opioidergic Drug Tramadol. Clin. Pharmacol. Ther. 90 (1), 143–150. 10.1038/clpt.2011.56 21562485

[B41] Tzvetkov M. V. Seitz T. Bokelmann K. Mueller T. Brockmöller J. Koepsell H. (2014). Does the Haplotype Met408-Del420, Which Was Apparently Predictive for Imatinib Efficacy, Really Exist and How Strongly May it Affect OCT1 Activity? Blood 123 (9), 1427–1429. 10.1182/blood-2013-11-535864 24578499

[B42] Vaidya S. Ghosh K. Shanmukhaiah C. Vundinti B. R. (2015). Genetic Variations of hOCT1 Gene and CYP3A4/A5 Genes and Their Association with Imatinib Response in Chronic Myeloid Leukemia. Eur. J. Pharmacol. 765, 124–130. 10.1016/j.ejphar.2015.08.034 26300393

[B43] Wang D.-S. Jonker J. W. Kato Y. Kusuhara H. Schinkel A. H. Sugiyama Y. (2002). Involvement of Organic Cation Transporter 1 in Hepatic and Intestinal Distribution of Metformin. J. Pharmacol. Exp. Ther. 302 (2), 510–515. 10.1124/jpet.102.034140 12130709

[B44] Wang L. Giannoudis A. Lane S. Williamson P. Pirmohamed M. Clark R. (2008). Expression of the Uptake Drug Transporter hOCT1 Is an Important Clinical Determinant of the Response to Imatinib in Chronic Myeloid Leukemia. Clin. Pharmacol. Ther. 83 (2), 258–264. 10.1038/sj.clpt.6100268 17568400

[B45] Wang L. Prasad B. Salphati L. Chu X. Gupta A. Hop C. E. C. A. (2015). Interspecies Variability in Expression of Hepatobiliary Transporters across Human, Dog, Monkey, and Rat as Determined by Quantitative Proteomics. Drug Metab. Dispos 43 (3), 367–374. 10.1124/dmd.114.061580 25534768

[B46] White D. L. Saunders V. A. Dang P. Engler J. Hughes T. P. (2010). OCT-1 Activity Measurement Provides a Superior Imatinib Response Predictor Than Screening for Single-Nucleotide Polymorphisms of OCT-1. Leukemia 24 (11), 1962–1965. 10.1038/leu.2010.188 20811406

[B47] White D. L. Saunders V. A. Dang P. Engler J. Zannettino A. C. W. Cambareri A. C. (2006). OCT-1-mediated Influx Is a Key Determinant of the Intracellular Uptake of Imatinib but Not Nilotinib (AMN107): Reduced OCT-1 Activity Is the Cause of Low In Vitro Sensitivity to Imatinib. Blood 108 (2), 697–704. 10.1182/blood-2005-11-4687 16597591

[B48] Zhang L. Dresser M. J. Gray A. T. Yost S. C. Terashita S. Giacomini K. M. (1997). Cloning and Functional Expression of a Human Liver Organic Cation Transporter. Mol. Pharmacol. 51 (6), 913–921. 10.1124/mol.51.6.913 9187257

[B51] Zhang J. Kobert K. Flouri T. Stamatakis A. (2014). PEAR: a fast and accurate Illumina Paired-End reAd mergeR. Bioinformatics 30, 614–620. 10.1093/bioinformatics/btt593 24142950 PMC3933873

