# Supplementary material for: Effects of a Common Eight Base Pairs Duplication at the Exon 7-Intron 7 Junction on Splicing, Expression, and Function of OCT1
Source: Front Pharmacol. 2021 May 7;12:661480. doi: 10.3389/fphar.2021.661480 (PMC8137991; doi:10.3389/fphar.2021.661480)
Supplement: Supplementary file 1 [file DataSheet1.docx]

Supplementary Material

Supplementary table 1: Used primers

|  | | | Sequence |
| --- | --- | --- | --- |
| Generation of an alternatively spliced OCT1 expression plasmid | | | |
| primer pair 1 | for | 5’-TAT CTC ACC TGG TAA GTT GAC CTG CAC TGG TTA A-3’ | |
|  | rev | 5’-TTA ACC AGT GCA GGT CAA CTT ACC AGG TGA GAT A-3’ | |
| Generation of minigene constructs | | | |
| primer pair 2 | for | 5’-AGG GCT TGG TGC AGA ACC AGA CAT T-3’ | |
|  | rev | 5’-ATT ACA GGA TAT CGC CAC CAA GCC CGG CTA ATT TTG TA-3’ | |
| primer pair 3 | Met408 for | 5‘-CAT CTA CCC CAT GGC C**A**T GTC AAA TTT GTT GGC-3‘ | |
|  | Met408 rev | 5‘-GCC AAC AAA TTT GAC A**T**G GCC ATG GGG TAG ATG-3‘ | |
| primer pair 4 | Val 408 for | 5‘-CAT CTA CCC CAT GGC C**G**T GTC AAA TTT GTT GGC-3‘ | |
|  | Val 408 rev | 5‘-GCC AAC AAA TTT GAC A**C**G GC CAT GGG GTA GAT G-3‘ | |
| PCR amplification of spliced exon 7 variants | | | |
| primer pair 5 | for | 5’- ATC TCA GTG GTA TTT GTG AG-3‘ | |
|  | rev | 5’- TCT GAG TCA CCT GGA CAA C-3’ | |
| SNaPshot primer | | | |
| primer 6 | rs35854239 | 5’-gatcgatcgaGGGAAATGATGAAAGCAGACAACTTACCA-3’ | |
| Primer Pyrosequencing | | | |
| primer pair 7 | minigene for | 5’- ATC TCA GTG GTA TTT GTG AG-3’ | |
|  | minigene rev | 5’- [BIO]CCT GCC TCG TCA TGA TTT TT-3’ | |
| primer pair 8 | liver for | 5’- CCT GCC TCG TCA TGA TTT TTA TC-3’ | |
|  | liver rev | 5’-[BIO] CAC TTC GAT TGC CTG GGA AA-3’ | |
| primer 9 | minigene seq | 5’- CTC GGG AGA TCT CCA-3’ | |
| primer 10 | liver seq | 5’- GAT GTT TAA CCA GTG CAG-3’ | |
| Next generation sequencing | | | |
| primer pair 11 | liver for | 5‘- [N] GGC AGC CTG CCT CGT CAT GAT-3‘ | |
|  | liver rev | 5‘- [N] CAG GCC CAA CAC CGC AAA CAA-3‘ | |
| primer pair 12 | minigene_for | 5‘- [N] CCT GCC TCG TCA TGA TTT TT-3‘ | |
|  | minigene_rev | 5‘- [N] ATC TCA GTG GTA TTT GTG AG-3‘ | |
| primer pair 13 | AEI for | 5‘-ACC AGC GGG AAC CTC TAC CTG GAT T-3‘ | |
|  | AEI mRNA rev | 5‘-CAT CAC TCC GAG GTT CCT GAC GAA T-3‘ | |
| primer pair 14 | AEI for | 5‘-ACC AGC GGG AAC CTC TAC CTG GAT T-3‘ | |
|  | AEI-gDNA rev | 5‘-TGT GCA CGG CCC CTC AAT TTA AAA T-3‘ | |

For, forward; rev, reverse; AEI, allelic expression imbalance; [BIO], biotinylation; [N], random nucleotide; Affected codons are underlined and mutated bases are highlighted in bold

**Additional information concerning primers used for next-generation sequencing:** Primers contained a 33 bp long overhang adapter sequence that allowed the addition of unique indices to each sample. To introduce a higher diversity to low complexity samples, the overhang adapter sequence was separated from the primer by zero to two random nucleotides [N] and a primer pool of overhang adapter primers with variable length was used.


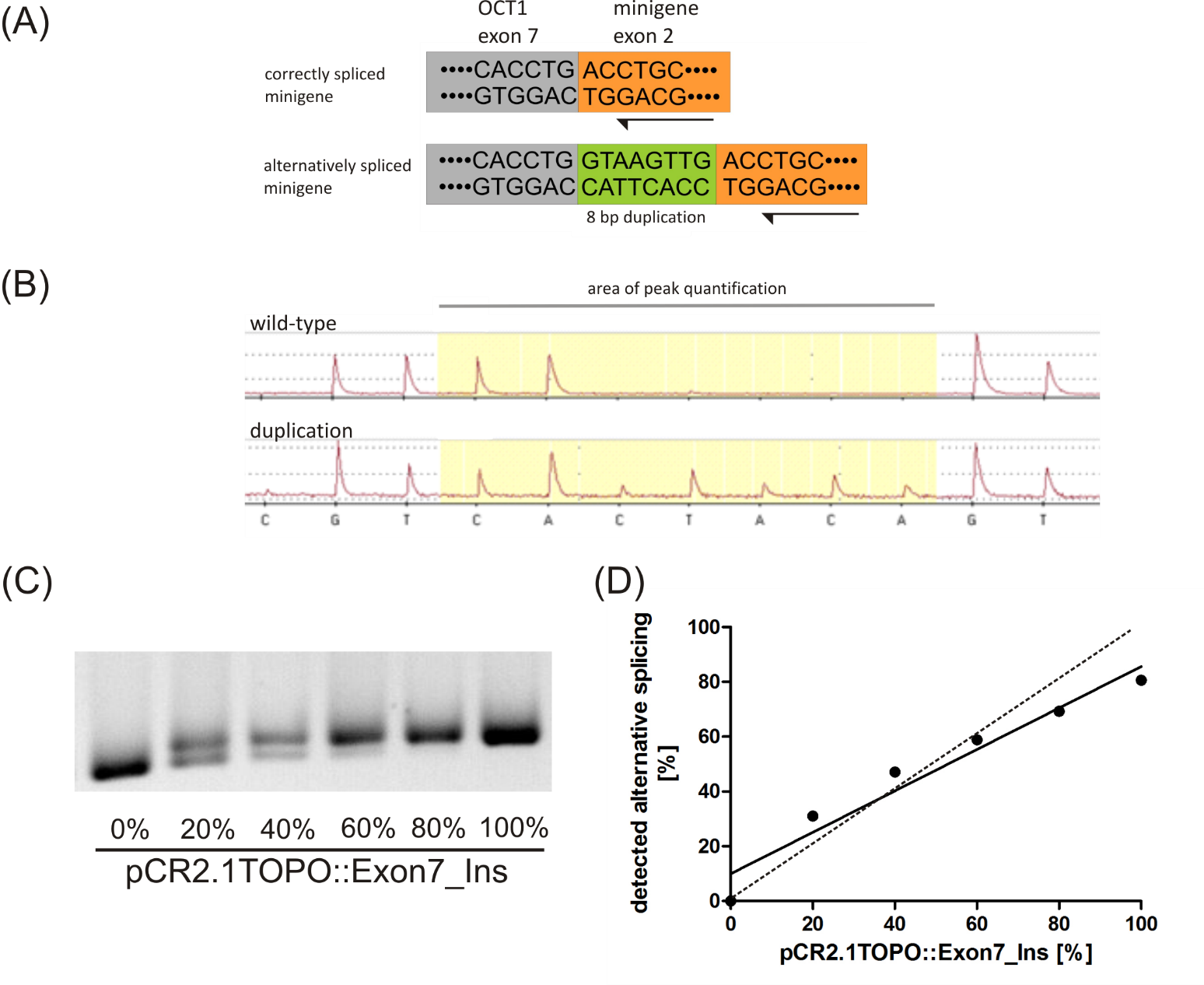


Supplementary Figure 1: Schematic representation of the pyrosequencing strategy used for quantitative analyses of the alternative splicing of the minigene. (A) Pyrosequencing analysis was performed using the sense DNA strand as template for sequencing by synthesis. The sequencing primer (indicated as black arrow) annealed in proximity of the 5’ end of minigene exon 2 allowing a reverse sequencing of the 8 bp duplication. The percentage between samples containing the 8 bp insertion (alternatively spliced) or without (correctly spliced) was determined by analyzing the resulting peak heights in the pyrogram trace. (B) Representative pyrograms of correctly spliced minigene (wild-type, upper panel) and partially alternatively spliced minigene vector (duplication, lower panel). (C) Representative gel of reference vector pCR2.1TOPO::Exon7_Ins (alternatively spliced) and pCR2.1TOPO::Exon7_Del (correctly spliced) in different ratios. (D) Calibration curve resulting from pyrosequencing of reference vectors in different ratios (bold), expected calibration curve (dotted).

To validate the pyrosequencing method, control vectors were generated. To this end, the minigene carrying the duplication allele was transfected into Huh7 cells and the splice products were amplified using the primers 5’-TCT GAG TCA CCT GGA CAA C-3’ forward and 5’-ATC TCA GTG GTA TTT GTG AG-3‘ reverse. Prior to cloning, 3‘ A overhangs were added to the amplicons. For this, 47 µl PCR product was incubated with 5 µl 5xOneTaq Standard Reaction Buffer, 1 µl dATP (10mM) and 1 µl OneTaq HotStart DNA polymerase at 72 °C for 15 min. The reaction was stopped by incubation on ice. Subsequently, amplicons were cloned into the pCR2.1-TOPO vector using the TOPO TA Cloning Kit (Invitrogen, Darmstadt, Germany). Cloning products were transformed into electrocompetent OneShot® TOP10 *E.coli.* After plating on kanamycin agar plates, single clones were picked, vectors isolated and the clones validated by capillary sequencing. One clone of each, correctly or alternatively spliced exon 7, was used for pyrosequencing validation. Different ratios of duplication and wild-type exon7 were quantified.

**Supplementary Table 2.** Summary of the exonic/splice variants identified in the *SLC22A1* gene in the 90 liver samples by next-generation exome sequencing, their allele frequencies and other annotations..

| **dbSNP  build 153** | **Position GRCh38.p12** | **Type** | **Alleles** | **Alt allele** | **Alt allele frequency (%)** | **HWE P value** | **Protein variant** | **cDNA variant** | **SLC22A1 mRNA** | | **OCT1 protein** | |
| --- | --- | --- | --- | --- | --- | --- | --- | --- | --- | --- | --- | --- |
|  |  |  |  |  |  |  |  |  | **Unadjusted P value** | **Holm-adjusted P value** | **Unadjusted P value** | **Holm-adjusted P value** |
| rs35888596 | 160122048 | missense | G/A | A | 1.1 | 1 | p.G38D | c.113G>A | 0.149 | 1 | 0.005 | 0.071 |
| rs1867351 | 160122091 | synonymous | T/C | C | 26.7 | 0.788 | p.S52S | c.156T>C | 0.434 | 1 | 0.623 | 1 |
| rs12208357 | 160122116 | missense | C/T | T | 9.4 | 1 | p.R61C | c.181C>T | 0.022 | 0.314 | **0.0002** | **0.003** |
| rs55918055 | 160122197 | missense | T/C | C | 0.6 | 1 | p.C88R | c.262T>C | 0.111 | 1 | 0.026 | 0.288 |
| rs761669870 | 160122251 | missense | C/A | A | 0.6 | 1 | p.L106M | c.316C>A | 0.253 | 1 | 0.293 | 1 |
| rs683369 | 160130172 | missense | G/C | C | 75.6 | 0.775 | p.L160F | c.480G>C | 0.121 | 1 | 0.107 | 0.854 |
| rs371130206 | 160136570 | synonymous | C/T | T | 0.6 | 1 | p.T327T | c.981C>T | 0.610 | 0.610 | 0.796 | 0.796 |
| rs2282143 | 160136611 | missense | C/T | T | 2.2 | 1 | p.P341L | c.1022C>T | 0.532 | 1 | 0.688 | 1 |
| rs34130495 | 160139792 | missense | G/A | A | 1.7 | 1 | p.G401S | c.1201G>A | 0.284 | 1 | 0.066 | 0.664 |
| rs628031 | 160139813 | missense | A/G | G | 55.0 | 0.674 | p.M408V | c.1222A>G | **0.0025** | **0.038** | 0.238 | 1.666 |
| rs72552763 | 160139849 | Indel_coding | ATG/- | - | 17.2 | 0.126 | p.M420del |  | 0.0653 | 0.783 | 0.080 | 0.718 |
| rs755828176 | 160139851 | Indel_coding | GA/- | - | 1.1 | 1 | p.M420Ilefs |  | 0.0475 | 0.618 | 0.730 | 1 |
| rs35854239 | 160139876 | Indel_splice | GTAAGTTG/- | - | 47.8 | 0.060 |  |  | **0.0014** | **0.022** | 0.019 | 0.251 |
| rs35956182 | 160143584 | missense | G/A | A | 0.6 | 1 | p.M440I | c.1320G>A | 0.569 | 1 | 0.782 | 1 |
| rs34059508 | 160154805 | missense | G/A | A | 3.9 | 1 | p.G465R | c.1393G>A | 0.376 | 1 | 0.025 | 0.301 |
| rs190524785 | 160158672 | 3' UTR | T/A | A | 1.1 | 1 |  |  | 0.513 | 1 | 0.018 | 0.247 |

Alt allele, alternate allele; HWE, Hardy-Weinberg equilibrium.

Boldface indicates significant P value after Holm correction.

Positions are based on GRCh38.p7 assembly of chromosome 6. Multivariate analysis of SLC22A1/OCT1 expression in relation to 16 *SLC22A1* genetic variants was performed with correction for 8 non-genetic covariates. P values are given either unadjusted or after adjusting for the number of variants according to Holm (Holm 1979). Multivariate statistical analyses were performed with the statistics software package R version 3.6.1 (http://www.r-project.org). To meet the Gaussian assumption of the statistical method, the mRNA expression data were transformed by taking the third root and the protein expression data were log-transformed. R-package SNPassoc-1.9-2 was applied to study associations between each genetic variant and SLC22A1/OCT1 mRNA/protein expression, considering the codominant genetic model and correcting for the 8 non-genetic factors sex, age, smoking habit, alcohol consumption, pre-surgery drugs, C-reactive protein, bilirubin, and γ-glutamyl transferase (Nies et al. 2009). Statistical significance was defined as P<0.05

References

Holm, Sture (1979): A Simple Sequentially Rejective Multiple Test Procedure. In: *Scandinavian Journal of Statistics* 6 (2), S. 65–70. Online verfügbar unter www.jstor.org/stable/4615733.

Nies, Anne T.; Koepsell, Hermann; Winter, Stefan; Burk, Oliver; Klein, Kathrin; Kerb, Reinhold et al. (2009): Expression of organic cation transporters OCT1 (SLC22A1) and OCT3 (SLC22A3) is affected by genetic factors and cholestasis in human liver. In: *Hepatology (Baltimore, Md.)* 50 (4), S. 1227–1240. DOI: 10.1002/hep.23103.
